# The origin and evolution of phototropins

**DOI:** 10.3389/fpls.2015.00637

**Published:** 2015-08-12

**Authors:** Fay-Wei Li, Carl J. Rothfels, Michael Melkonian, Juan C. Villarreal, Dennis W. Stevenson, Sean W. Graham, Gane K.-S. Wong, Sarah Mathews, Kathleen M. Pryer

**Affiliations:** ^1^Department of Biology, Duke UniversityDurham, NC, USA; ^2^University Herbarium and Department of Integrative Biology, University of California at BerkeleyBerkeley, CA, USA; ^3^Botany Department, Cologne Biocenter, University of CologneCologne, Germany; ^4^Royal Botanic Gardens EdinburghEdinburgh, Scotland; ^5^New York Botanical GardenBronx, NY, USA; ^6^Department of Botany, University of British ColumbiaVancouver, BC, Canada; ^7^Department of Biological Sciences, University of AlbertaEdmonton, AB, Canada; ^8^Department of Medicine, University of AlbertaEdmonton, AB, Canada; ^9^BGI-ShenzhenShenzhen, China; ^10^CSIRO, Centre for Australian National Biodiversity ResearchCanberra, ACT, Australia

**Keywords:** blue-light, convergent evolution, land plants, photoreceptors, phototropism

## Abstract

Plant phototropism, the ability to bend toward or away from light, is predominantly controlled by blue-light photoreceptors, the phototropins. Although phototropins have been well-characterized in *Arabidopsis thaliana*, their evolutionary history is largely unknown. In this study, we complete an in-depth survey of phototropin homologs across land plants and algae using newly available transcriptomic and genomic data. We show that phototropins originated in an ancestor of Viridiplantae (land plants + green algae). Phototropins repeatedly underwent independent duplications in most major land-plant lineages (mosses, lycophytes, ferns, and seed plants), but remained single-copy genes in liverworts and hornworts—an evolutionary pattern shared with another family of photoreceptors, the phytochromes. Following each major duplication event, the phototropins differentiated in parallel, resulting in two specialized, yet partially overlapping, functional forms that primarily mediate either low- or high-light responses. Our detailed phylogeny enables us to not only uncover new phototropin lineages, but also link our understanding of phototropin function in *Arabidopsis* with what is known in *Adiantum* and *Physcomitrella* (the major model organisms outside of flowering plants). We propose that the convergent functional divergences of phototropin paralogs likely contributed to the success of plants through time in adapting to habitats with diverse and heterogeneous light conditions.

## Introduction

Light is the ultimate source of energy for almost all of life on earth, and a remarkable diversity of organisms uses photosynthesis to convert light into metabolic energy. Many of these organisms have also evolved phototropic/phototactic responses, and those in plants are particularly sophisticated—involving movement of shoots, leaves, and/or chloroplasts—in order to optimize their photosynthetic capacity. Charles Darwin pioneered modern research on phototropism by demonstrating that the coleoptile tip is the point of light perception (Darwin, 1880). Darwin proposed that a transmissible substance produced at the tip is responsible for inducing phototropic movements in plants. This insight led to the first discovery of a plant hormone, auxin, and later to the identification of the blue-light photoreceptors for phototropism—phototropins (Briggs et al., 2001; Christie and Murphy, 2013; Briggs, 2014).

Phototropins regulate key physiological responses that are under light control, including positive phototropism of shoots, negative phototropism of roots, chloroplast accumulation, and avoidance, stomatal opening, leaf expansion, and seedling elongation (Christie, 2007). Our current understanding of the function and biochemistry of phototropins originates from basic research on *A. thaliana*, and to a lesser extent on *Adiantum capillus-veneris* (a fern) and *Physcomitrella patens* (a moss). Only a few studies have attempted to address the origin and evolution of phototropins (Briggs et al., 2001; Lariguet and Dunand, 2005; Galván-Ampudia and Offringa, 2007) and all were based on limited sequence samples. The orthology of phototropin genes has therefore been ambiguous, confounding assignments of functional homology, and impeding our understanding of how phototropin evolution has allowed plants to adapt to light environments.

An extraordinary phototropin derivative is neochrome, which possesses supplementary red/far-red-sensing domains from phytochromes (Nozue et al., 1998). In ferns, neochrome can sense both blue and red/far-red light to modulate chloroplast movement and phototropism (Kanegae et al., 2006; Kanegae and Kimura, 2015). We previously reconstructed a phototropin phylogeny with an aim to elucidate the origin of neochromes (Li et al., 2014). However, that phylogeny had insufficient taxon sampling to accurately infer broad patterns of phototropin evolution, including the position of key phototropin duplications.

For this study, we greatly expanded our search for phototropins in genomes and transcriptomes from across land plants, green algae, red algae, glaucophytes, cryptophytes, haptophytes, and stramenopiles (Supplementary Tables S1 and S2). Using these data, we reconstructed a detailed phylogeny of phototropins and examined patterns of gene duplication. Our results suggest that phototropins likely originated in an ancestor of Viridiplantae (land plants + green algae). By reviewing published phototropin functional studies in light of our new gene phylogeny, we determined that phototropin paralogs repeatedly underwent functional divergences. These were likely to be important for adapting to diverse and heterogeneous light environments through time.

## Materials and Methods

### Mining Phototropin Homologs from Transcriptomes and Genomes

We searched a total of 194 transcriptomes and 26 genomes (Supplementary Table S1). To mine phototropin homologs, we used the BlueDevil python pipeline following Li et al. (2014) for transcriptomes, and for genomes we used BLASTp implemented in Phytozome (Goodstein et al., 2012) or individual genome portals (Supplementary Tables S1 and S2). A phototropin sequence from *Anthoceros bharadwajii* [voucher: Chantanaorrapint 229 (PSU)] was obtained by PCR and cloning (primers: photF1970 and photR4102; Li et al., 2014).

### Sequence Alignment and Phylogenetic Reconstruction

We used MUSCLE (Edgar, 2004) with the default settings to align the amino acid sequences, and then back-translated these to nucleotides. The resulting alignment was manually improved based on known domain boundaries; unalignable regions were excluded prior to phylogenetic analyses. The final alignment length is 2,025 bp, within which most of the sequences are complete or near complete (Supplementary Figure S1).

We used PartitionFinder v1.1.1 (Lanfear et al., 2012) to obtain the optimal data partitioning scheme (by codon position) and the associated nucleotide substitution models (GTR + I+ Γ substitution model applied independently to the first, second, and third codon positions). Garli v2.0 (Zwickl, 2006) was employed to find the best maximum likelihood tree with “genthreshfortopoterm” set to 500,000 and eight independent runs from different random-addition starting trees. We carried out bootstrapping to assess branch support, using RAxML v8.1.11 (Stamatakis, 2006) with 1,000 replicates. The same partition scheme and models were used in MrBayes v3.2.3 (Ronquist et al., 2012) Bayesian inference. We carried out two independent MCMC runs, each with four chains and trees sampled every 1,000 generations (chain length: 6.451 × 10^9^ generations). We unlinked substitution parameters and set the rate prior to vary among subsets. The resulting MCMC statistics were inspected in Tracer (Rambaut and Drummond, 2013) to ensure convergence and proper mixing; 25% of the total generations were discarded as burn-in before compiling the majority consensus tree. The alignment and tree files are deposited in Dryad^1^.

### Target Enrichment for Confirming Phototropin Copy Number in Hornworts

The target enrichment data were from Li et al. (2015), whereby a hornwort (*Anthoceros punctatus*) DNA library was hybridized with 7,502 120mer RNA probes to enrich phototropin, phytochrome, and neochrome homologs. The probe sequences can be found in Dryad^2^. We used an enrichment protocol of Li et al. (2013), which can potentially capture sequences with similarity as low as 61%. The captured fragments were sequenced on one-tenth of a MiSeq (250 bp paired-end) run. The reads are deposited in NCBI SRA (SRP055877). We used Scythe v0.994 (Buffalo, 2014) to remove the adaptor sequences with the default prior contamination rate, and Sickle v1.33 (Joshi and Fass, 2011) to trim the low-quality bases with a quality threshold of 33. We assembled the processed reads using SOAPdenovo (Luo et al., 2012) with kmer of 33, 63, and 93, and used CAP3 (Huang, 1999) to merge the three assemblies from different kmer sizes. The phototropin contigs were identified by BLASTn (Camacho et al., 2009).

## Results

### The Origin of Phototropins

**FIGURE 1:**
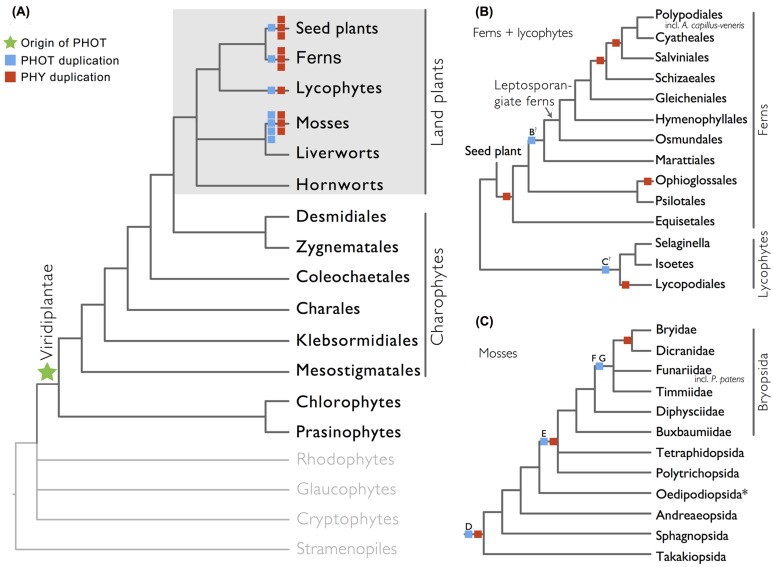
**Organismal lineages screened for phototropin homologs. (A)** Viridiplantae and algae. Lineages that lack phototropin are depicted in gray. Topology derived from Burki et al. (2012) and Wickett et al. (2014). Phototropin (PHOT) and phytochrome (PHY) duplications are only shown on land plant branches (within gray box). **(B)** Ferns and lycophytes; topology derived from Rothfels et al. (2015). **(C)** Mosses; topology derived from Cox et al. (2010). Capital letters above blue squares denote phototropin duplication events mentioned in the text and in **Figures **2**–4**. “?” indicates that the exact phylogenetic position of the gene duplication event is ambiguous. “^∗^” indicates that the lineage was not sampled.

We show here that phototropins are present in all major land plant lineages (seed plants, ferns, lycophytes, mosses, liverworts, and hornworts), as well as in green algae (charophytes, chlorophytes, and prasinophytes; **Figure 1A**). In contrast, we did not recover phototropins from glaucophytes, red algae (rhodophytes), cryptophytes, haptophytes, or stramenopiles, indicating that the origin of phototropin most likely took place in a common ancestor of Viridiplantae (green algae + land plants; **Figure 1A**).

### Phototropin Phylogeny

**FIGURE 2:**
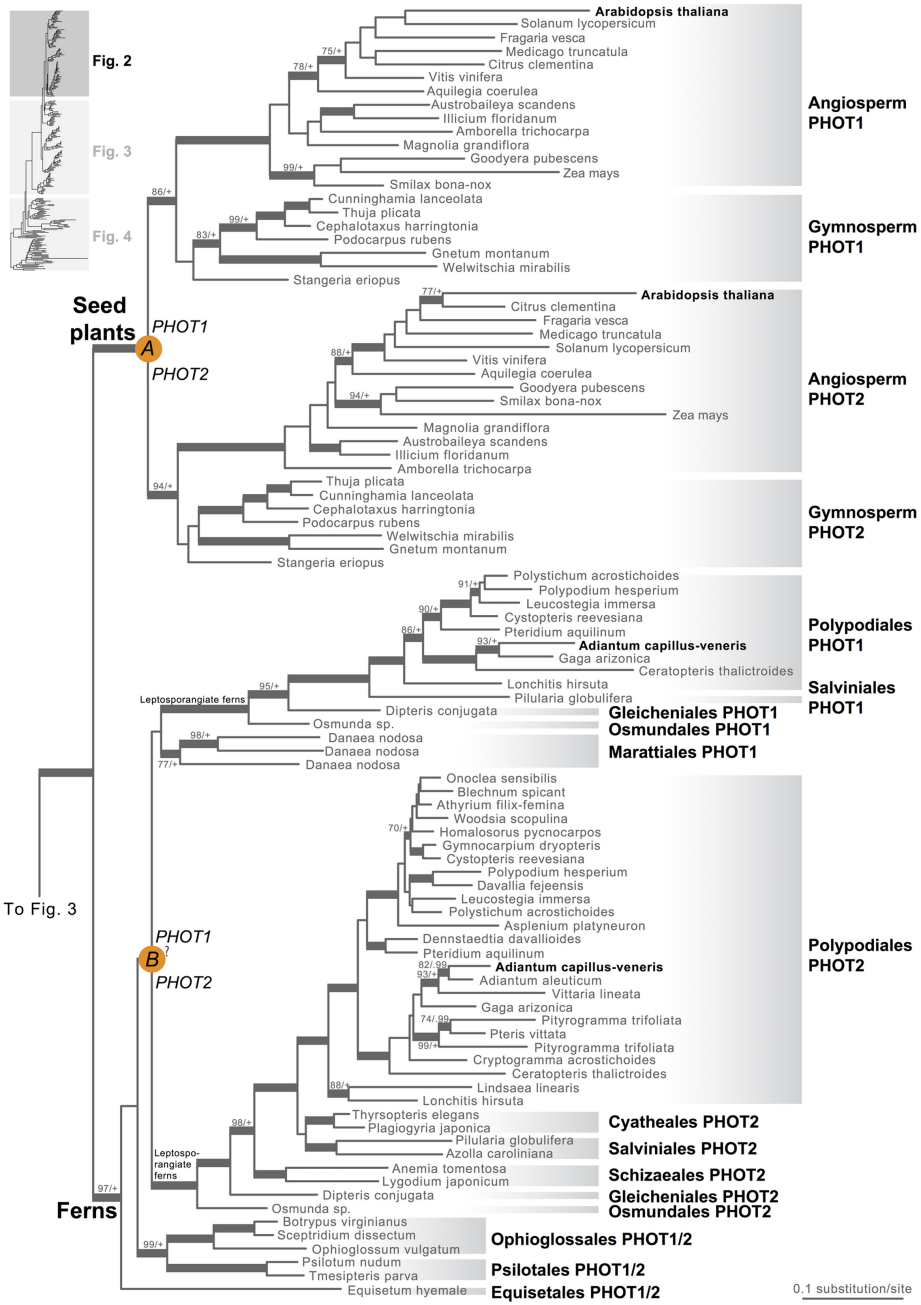
**Phylogenetic relationships of seed plant and fern phototropins**. The phylogeny tree continues to **Figures 3** and **4**. Orange circles indicate inferred phototropin (PHOT) duplication events. The italicized capital letter within each circle corresponds to the duplication event mentioned in the text, and the numbers/letters adjacent to each orange circle are the names of the gene duplicates. Support values associated with branches are maximum likelihood bootstrap values (BS)/Bayesian posterior probabilities (PP); these are only displayed (along with thickened branches) if BS > 70 and PP > 0.95. “+” denotes BS = 100 or PP = 1.00. Thickened branches without numbers are 100/1.0. “?” indicates that the exact phylogenetic position of the gene duplication event is ambiguous.

**FIGURE 3:**
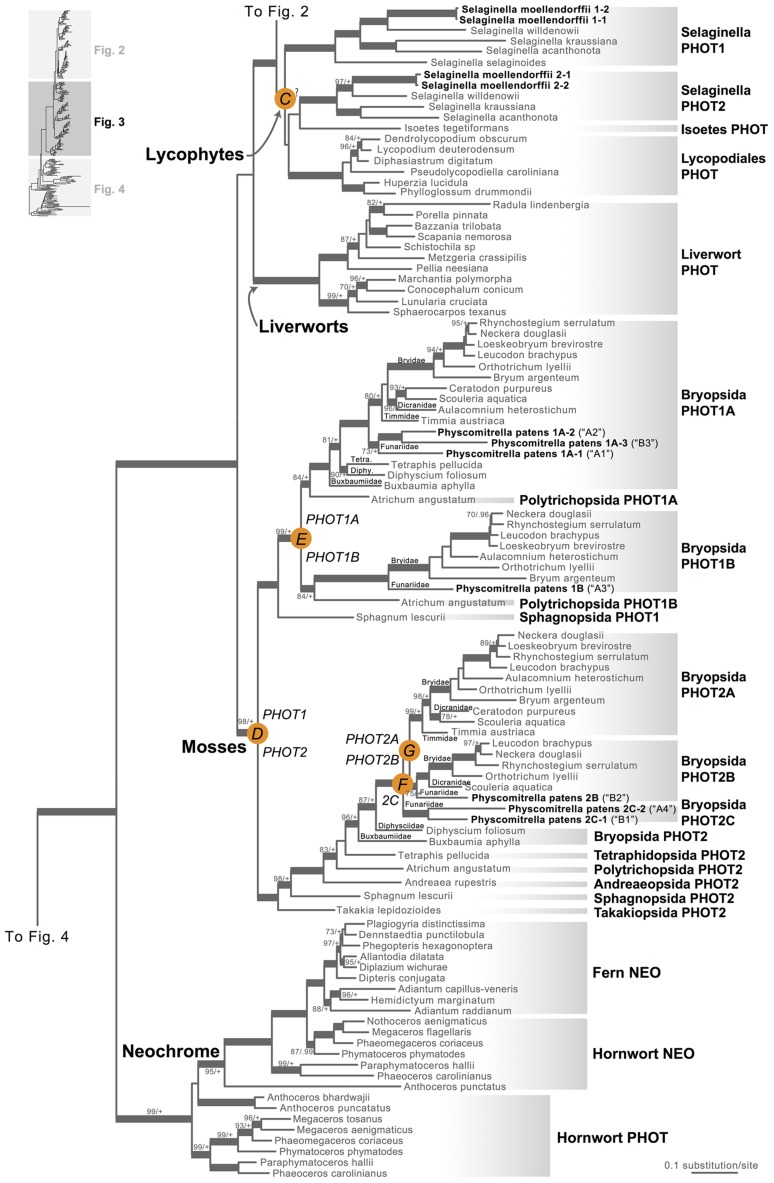
**Phylogenetic relationships of lycophyte and bryophyte phototropins**. The phylogeny tree is continued from **Figure 2**. Previous gene annotations for *Physcomitrella patens* are in parentheses. Orange circles indicate inferred phototropin (PHOT) duplication events. The italicized capital letter within each circle corresponds to the duplication event mentioned in the text, and the numbers/letters adjacent to each orange circle are the names of the gene duplicates. Support values associated with branches are maximum likelihood bootstrap values (BS)/Bayesian posterior probabilities (PP); these are only displayed (along with thickened branches) if BS > 70 and PP > 0.95. “+” denotes BS = 100 or PP = 1.00. Thickened branches without numbers are 100/1.0. “?” indicates that the exact phylogenetic position of the gene duplication event is ambiguous.

Our phototropin phylogeny is largely congruent with published organismal relationships (Forrest et al., 2006; Cox et al., 2010; Gontcharov and Melkonian, 2010; Villarreal and Renner, 2012; Wickett et al., 2014; Rothfels et al., 2015). Seed–plant phototropins form a monophyletic group that is sister to fern phototropins (**Figure 2**). Here we infer a single gene duplication event in seed plants, leading to *A. thaliana AtPHOT1*, and *AtPHOT2*. Because seed-plant *PHOT1* and *PHOT2* clades each include sequences from angiosperms and gymnosperms, the duplication event that gave rise to these two homologs predates the divergence of angiosperms from the ancestors of extant gymnosperms (“A” in **Figure 2**). We also find strong evidence for the monophyly of fern phototropins (**Figure 2**). Leptosporangiate ferns have two phototropin homologs that we designate fern *PHOT1* and *PHOT2*, in reference to *A. capillus-veneris AcPHOT1* and *AcPHOT2* (Kagawa et al., 2004), respectively. The earliest-diverging fern lineages, Equisetales, Psilotales, and Ophioglossales, each have one phototropin gene, representing the pre-duplicated version of fern *PHOT1* and *PHOT2*. The exact phylogenetic position of the split of fern *PHOT1* and *PHOT2* is ambiguous due to a lack of branch support, although it probably was prior to the most recent common ancestor of leptosporangiate ferns and Marattiales (“B” in **Figures 1B** and **2**). We also infer a single duplication event in the lycophyte *Selaginella*, leading to *Selaginella PHOT1* and *PHOT2* based on the genome annotation of *S. moellendorffii* (Banks et al., 2011). The phylogenetic position of this duplication is unclear (“C” in **Figure 3**), but it must predate the common ancestor of extant *Selaginella* because the *PHOT1* clade contains all known major *Selaginella* lineages (Korall and Kenrick, 2002). For Isoetales and Lycopodiales, we found only one phototropin homolog, but determining whether it is indeed a single-copy gene in these lineages will require confirmation with additional data.

All liverwort transcriptomes we examined contained a single phototropin (**Figure 3**), a result consistent with the recent demonstration that phototropin in *Marchantia polymorpha* is a single-copy gene (Komatsu et al., 2014). Hornwort phototropins also appear to be single-copy based on our screening of hornwort transcriptomes and a low-coverage genome draft of *Anthoceros punctatus* (Li et al., 2014). To further investigate gene copy number in hornworts, we used a target-enrichment strategy to sequence all phototropin-, phytochrome- and neochrome-like genomic fragments in *Anthoceros punctatus*. We found no additional divergent phototropin copies, and recovered one phytochrome and one neochrome gene copy.

**FIGURE 4:**
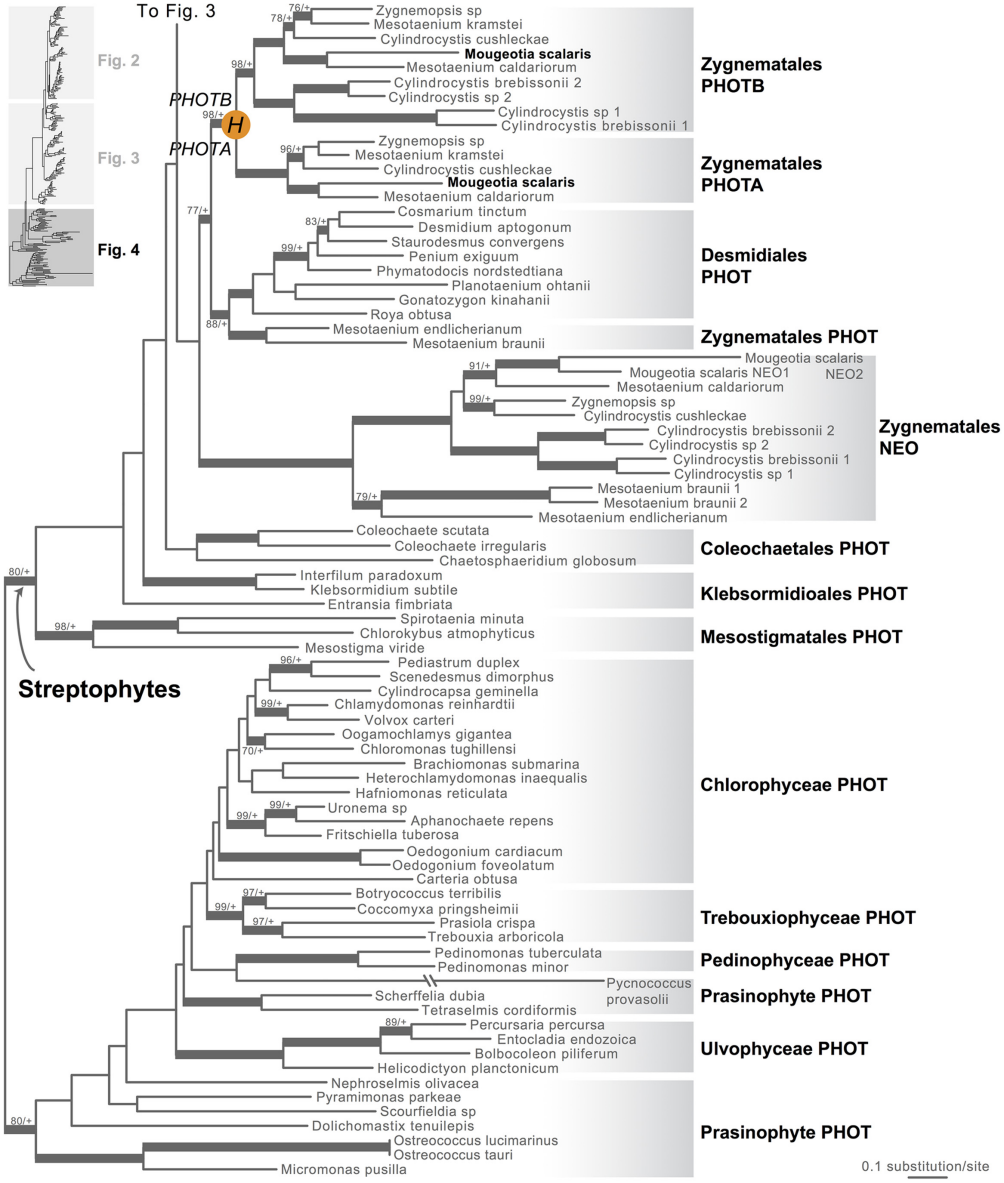
**Phylogenetic relationships of algal phototropins**. The phylogenetic tree is continued from **Figure 3**. Orange circles indicate inferred phototropin (PHOT) duplication events. The italicized capital letter within each circle corresponds to the duplication event mentioned in the text, and the numbers/letters adjacent to each orange circle are the names of the gene duplicates. Support values associated with branches are maximum likelihood bootstrap values (BS)/Bayesian posterior probabilities (PP); these are only displayed (along with thickened branches) if BS > 70 and PP > 0.95. “+” denotes BS = 100 or PP = 1.00. Thickened branches without numbers are 100/1.0.

Moss phototropins, on the other hand, have a significantly more complex evolutionary history (**Figures 1C** and **3**). We discovered that the published phototropin annotations from the moss *P. patens* genome (Rensing et al., 2008) do not correctly reflect gene orthology. Because “*PHOTAs*” and “*PHOTBs*” are intermingled, we reclassified the moss phototropins based on the phylogenetic relationships inferred here (**Table 1**, **Figure 3**). Prior to the initial divergences among extant mosses, a gene duplication (“D” in **Figures 1C** and **3**) gave rise to moss *PHOT1* and *PHOT2*. In moss *PHOT1*, a second duplication occurred in a common ancestor of Bryopsida and Polytrichopsida (“E” in **Figures 1C** and **3**) that split moss *PHOT1* into moss *PHOT1A* and *PHOT1B*. In moss *PHOT2*, two additional duplications occurred (“F” and “G” in **Figures 1C** and **3**) subsequent to the divergence of Diphysciidae (Bryopsida), resulting in moss *PHOT2A-C*. Both moss *PHOT2A* and *PHOT2B* are present in Dicranidae and Bryidae, whereas moss *PHOT2C* is only known in *P. patens* (Funariidae). *P. patens* may also have lost the *PHOT2A* homolog. Alternatively, because the placement of *PHY2C* is not supported, these *P. patens* sequences could belong to *PHY2A*, requiring only one gene duplication that resulted in moss *PHY2A* and *PHY2B*. Most green algal transcriptomes and genomes revealed a single phototropin gene (**Figure 4**), with the exception of those from Zygnematales, where two phototropin homologs are present (*PHOTA* and *PHOTB*).

**Table 1:** Reclassification of *Physcomitrella patens* phototropins based on gene orthology.

Proposed new name	Previous annotation	Genbank accession
*PpPHOT1A-1*	*PpPHOTA1*	XM_001774204
*PpPHOT1A-2*	*PpPHOTA2*	XM_001774562
*PpPHOT1A-3*	*PpPHOTB3*	XM_001755269
*PpPHOT1B*	*PpPHOTA3*	XM_001765356
*PpPHOT2B*	*PpPHOTB2*	XM_001785674
*PpPHOT2C-1*	*PpPHOTB1*	XM_001766357
*PpPHOT2C-2*	*PpPHOTA4*	XM_001763052

### All Algal Neochromes Lack the Conserved Cysteine Residue at the LOV2 Domain

Neochrome (neo, **Figures 3** and **4**) is a unique chimeric phototropin variant that possesses supplementary red/far-red-sensing domains from phytochromes (Nozue et al., 1998). Recent studies have revealed two independent origins of neochromes, one in zygnematalean algae and the other in hornworts (Suetsugu et al., 2005; Li et al., 2014). Interestingly, the neochromes found in ferns were determined to be derived from hornworts via horizontal gene transfer (Li et al., 2014). Neochrome perceives both blue and red/far-red light to mediate phototropic responses in ferns (Kawai et al., 2003; Kanegae et al., 2006; Kanegae and Kimura, 2015), and it appears to have played a significant role in their diversification (Schneider et al., 2004; Schuettpelz and Pryer, 2009). The function of neochrome in hornworts and zygnematalean algae, however, remains unclear. Because some zygnematalean algae have plate-like chloroplasts that rotate in response to both blue and red/far-red light irradiation (Haupt and Scheuerlein, 1990), it was hypothesized that algal neochrome, originally discovered in *Mougeotia scalaris*, is the candidate gene responsible for this movement (Suetsugu et al., 2005). However, neochrome in *M. scalaris* responds only to red/far-red light and not to blue light (Suetsugu et al., 2005; Kagawa and Suetsugu, 2007).

**FIGURE 5:**
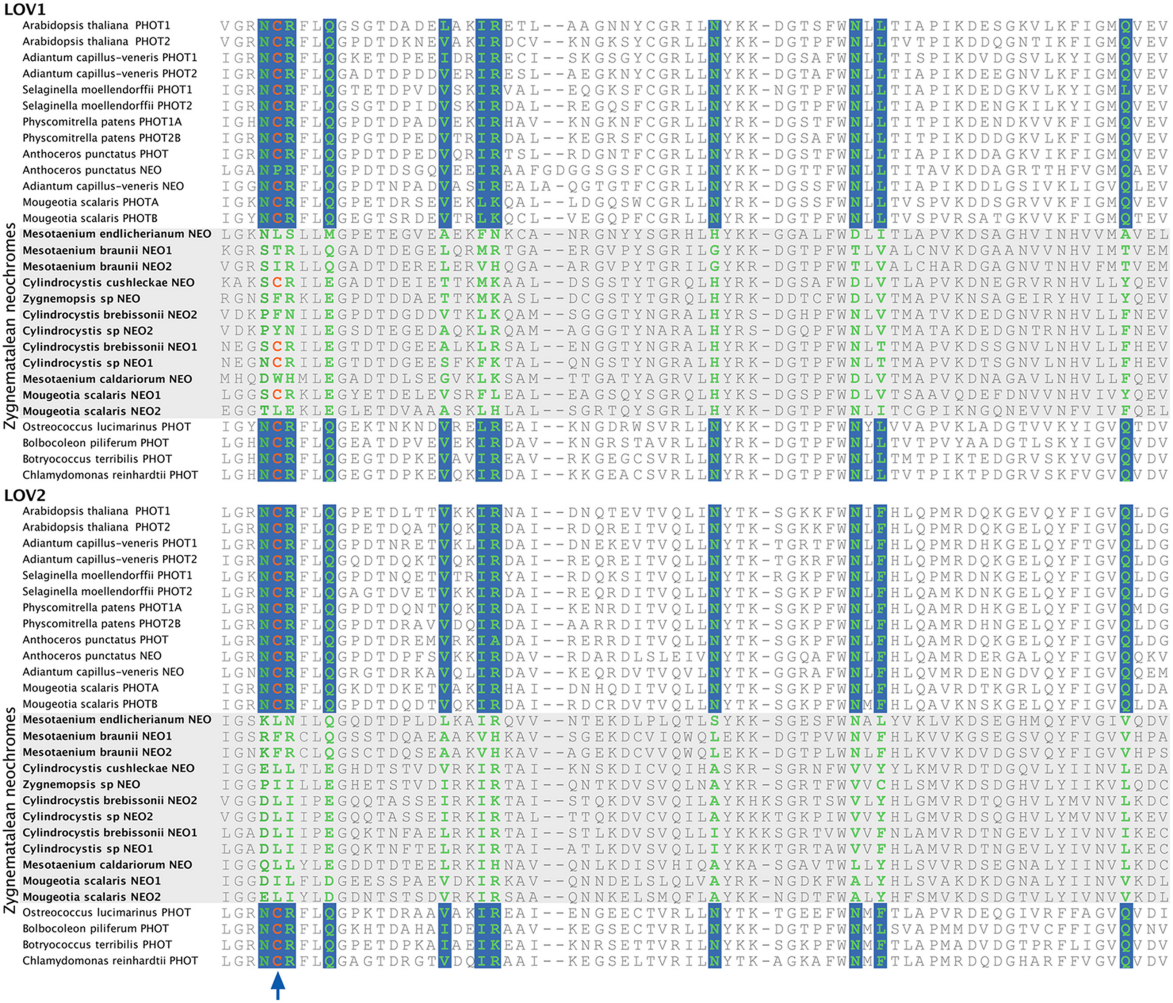
**Alignment of a portion of LOV1 and LOV2 domains in selected phototropins and neochromes**. The site for flavin mononucleotide (FMN) adduct formation is marked by an arrow, and the FMN-interacting sites are shown in green with a blue background. All zygnematalean neochromes (highlighted in a gray box) lack the conserved cysteine residue in the LOV2 domain, and several residues that interact with FMN are also not conserved.

To explore whether *M. scalaris* might be anomalous among zygnematalean algae in having a neochrome that is not responsive to blue light, we examined all the algal neochromes that we recovered. As is the case with the neochrome of *M. scalaris*, none has the conserved cysteine residue in the LOV2 domain (**Figure 5**) that is essential for the formation of flavin mononucleotide (FMN) chromophore adduct and blue-light signal transduction (Christie, 2007; but see Kanegae and Kimura, 2015). Furthermore, many of the FMN-interacting residues (Crosson and Moffat, 2001) are also not conserved in zygnematalean neochromes (**Figure 5**). It is thus possible that all zygnematalean algae use neochromes only for sensing red/far-red light, and use other blue-light photoreceptors (most likely phototropins; Kagawa and Suetsugu, 2007; Banas et al., 2012) to maneuver chloroplast rotations. However, Kanegae and Kimura (2015) recently discovered that in fern neochromes, substitution of the cysteine residue did not completely abolish blue-light-induced phototropism. They further suggested that the phytochrome chromophore, phytochromobilin, could have the capacity to perceive blue light and then relay the signals. Therefore, we cannot rule out the possibility that some zygnematalean neochromes can sense blue light through a FMN-independent mechanism.

## Discussions

### A New Phototropin Gene Orthology

With a detailed phototropin phylogeny that encompasses all of green plant representatives, we were able to discover new phototropin lineages and pinpoint the timing of gene duplications (**Figures 1–4**). This new understanding of phototropin gene orthology refutes the previous assertion that the “*PHOT2*” ortholog is the ancestral phototropin and that “*PHOT1*” evolved later in seed plants (Galván-Ampudia and Offringa, 2007); the ancestral phototropin is neither “*PHOT1*” nor “*PHOT2*.” Rather, a single *PHOT* ancestral sequence gave rise to the multiple gene copies found in seed plants, lycophytes, ferns, mosses, and zygnematalean algae (**Figures 1–4**). Independent duplications occurred subsequently, such that *PHOT1* and *PHOT2* in each of these plant lineages are more closely related to one another than they are to other *PHOT1*s or *PHOT2*s.

### Convergent Sub-Functionalization of Phototropins

Our findings on gene orthology also have important implications for understanding the functional evolution of phototropins. Plants often respond differently under low- and high-light levels; chloroplasts, in particular, accumulate on the periclinal walls under weak light, but retreat to anticlinal walls when the light intensity is too high. Our phylogenetic reconstruction suggests that phototropins repeatedly duplicated and diverged, and that after doing so, they subsequently specialized in mediating either low– or high-light responses, although functional redundancies do exist (Christie, 2007). Of the two phototropins known in *A. thaliana*, Atphot1 mediates phototropism under low light intensity, and is more sensitive than Atphot2 in triggering chloroplast accumulation (Sakai et al., 2001). Atphot2, in contrast, responds predominantly to high-light intensity, and is primarily responsible for chloroplast avoidance under strong light (Kagawa et al., 2001; Luesse et al., 2010). A similar functional differentiation can also be seen in the fern *A. capillus-veneris* Acphot1 and Acphot2. Acphot2 controls chloroplast avoidance under high-light intensity, whereas Acphot1 has little or no role in this response (Kagawa et al., 2004). Similarly, in the moss *P. patens*, Ppphot1A-2 (see **Table 1**) is the primary mediator for the chloroplast avoidance response, and plays a redundant role in the accumulation behavior (Kasahara et al., 2004).

The single phototropin in the liverwort *M. polymorpha* can respond to a wide range of light intensities and triggers both chloroplast avoidance and accumulation responses (Komatsu et al., 2014). Because liverworts represent one of the deepest splits in land-plant phylogeny (Wickett et al., 2014), their unspecialized phototropins suggest that the ancestral land plant phototropin was probably a “general-purpose” photoreceptor. The subsequent and parallel specializations of phototropin into forms that are responsive to low–, or high-light intensities may have played an important role in the adaptation of early land plants to Earth’s changing landscapes. From the formation of the earliest forests by cladoxylopsid ferns about 385 million years ago (Stein et al., 2007) through to today’s angiosperm-dominated terrestrial ecosystems, light environments have become increasingly complex, and deeply shaded habitats have expanded. Possessing duplicated phototropin genes dedicated to functioning under different light intensities would have been advantageous (Galen et al., 2004) compared with having a single, general-purpose phototropin. The fact that land plant lineages with duplicated phototropins (seed plants, leptosporangiate ferns and mosses) are more species-rich than those without (liverworts, hornworts, and non-leptosporangiate ferns) is consistent with such an advantage, although many other traits could also have contributed to this disparity in diversity.

Compared to land-plant phototropins, much less is known about the function of algal phototropins, where most of the research done on *Chlamydomonas reinhardtii* shows that phototropins regulate sexual processes (Huang and Beck, 2003), eyespot size and phototactic behavior (Trippens et al., 2012). Interestingly, the phototropin gene from *C. reinhardtii* can partially rescue *A. thaliana phot1 phot2* double mutant phenotypes, suggesting that the phototropin signal transduction pathway is deeply conserved from green algae to seed plants (Onodera et al., 2005). Future studies focusing on phototropins across more algal lineages (**Figure 4**) should help to clarify early phototropin evolution in unicellular organisms, and the genetic basis of its functional differentiation in land plants.

### Patterns of Phototropin Copy Expansion and Stasis Resemble that of Phytochromes

The evolutionary pattern that we observe here for phototropins shows a striking resemblance to that for phytochromes. Both photoreceptors (phytochromes and phototropins) duplicated repeatedly in seed plants, ferns, lycophytes, and mosses, while remaining single-copy in liverworts and hornworts (**Figure 1**; Li et al., 2015). Although this pattern of concerted gene family expansion and stasis could be due to whole genome duplications (WGD), the exact evolutionary positions of gene duplication events in these two photoreceptors differ—they did not all happen along the same phylogenetic branches (**Figure 1**), suggesting that WGD is not solely responsible. Recent studies have shown that phototropins and phytochromes not only share cross-talk in their signal transduction pathways (Lariguet et al., 2006; de Carbonnel et al., 2010; Demarsy et al., 2012), but also can physically interact (Jaedicke et al., 2012). The extent to which phototropins and phytochromes are co-evolving would be an interesting topic for future research.

## Conclusion

In summary, we have leveraged recent genomic and transcriptomic data to discover phototropins from across a broad sample of photosynthetic eukaryotes. Our study reveals that phototropins are unique to Viridiplantae, and that gene family expansion and stasis have operated uniquely within each of the various land plant lineages, a pattern similar to that of phytochromes (Li et al., 2015). Existing functional data for phototropins, interpreted in light of our gene phylogeny, suggest a history of repeated gene duplications followed by parallel functional divergences. Our broad phylogenetic approach is an important complement to ongoing photobiological research focused on a small number of plant model organisms, and will enable new research linking ecology, evolution, and photochemistry to understanding how plants adapt (and have adapted) to variable light environments.

## Conflict of Interest Statement

The authors declare that the research was conducted in the absence of any commercial or financial relationships that could be construed as a potential conflict of interest.

## Footnotes

1 http://dx.doi.org/10.5061/dryad.321bv.

2 http://dx.doi.org/10.5061/dryad.5rs50.
