# Supplementary material for: The origin and evolution of phototropins
Source: Front Plant Sci. 2015 Aug 12;6:637. doi: 10.3389/fpls.2015.00637 (PMC4532919; doi:10.3389/fpls.2015.00637)
Supplement: Supplementary file 3 [file Image_1.PDF]

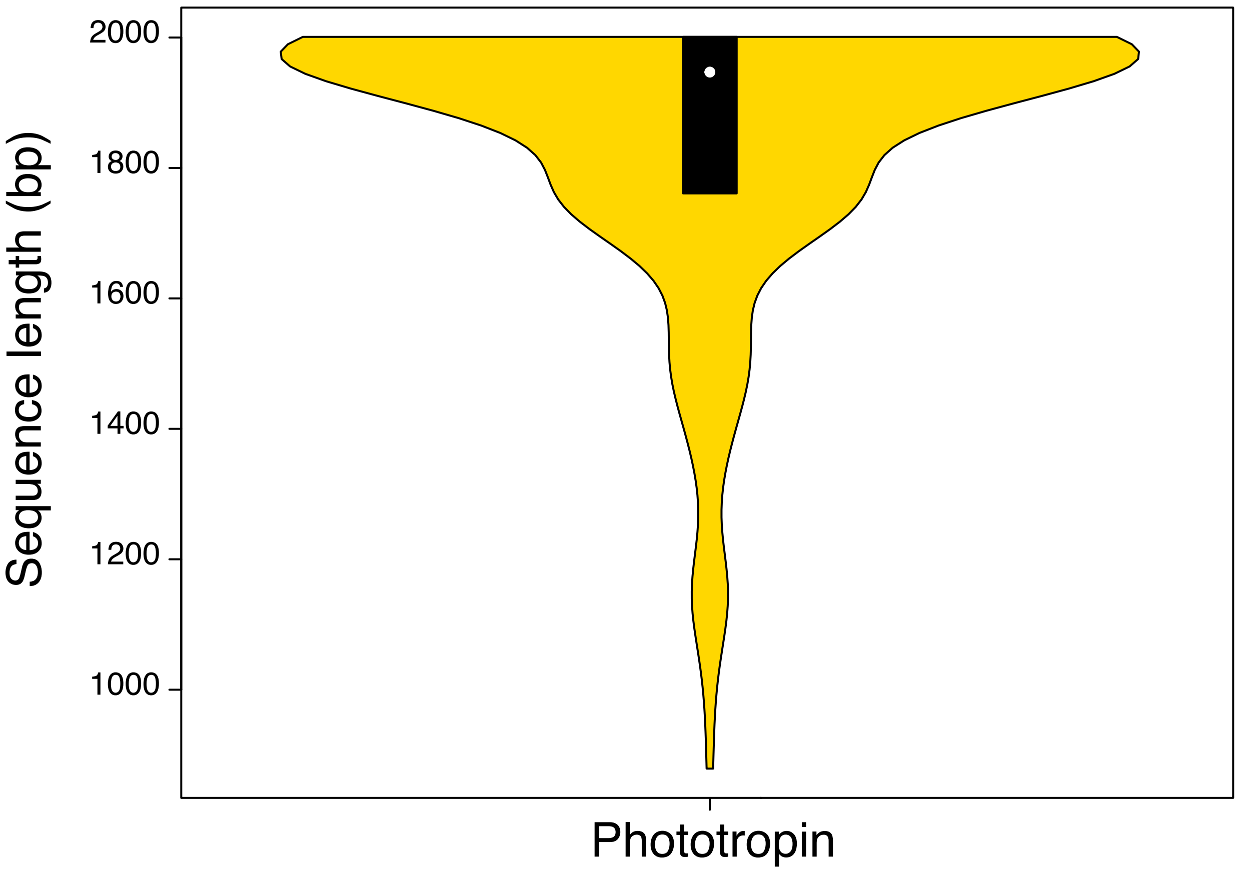

Figure S1. **A violin-plot showing sequence length distribution in the phototropin alignment.** The alignment is 2025 bp long, and the majority of the sequences are complete or near complete. The spread along the Y-axis is sequence length and along the X-axis is density. The white circle indicates the medium and the black box represents the quartile range.
